# A Two-Stage ESM-Based Machine Learning Pipeline for Robust Hierarchically Evaluated Enzyme Function Prediction

**DOI:** 10.34133/csbj.0246

**Published:** 2026-10-29

**Authors:** Xiao Hua, Ghjuvan M. Grimaud

**Affiliations:** Division of Biotechnology and Applied Microbiology, Department of Process and Life Science Engineering, Faculty of Engineering, Lund University, SE-221 00 Lund, Sweden.

## Abstract

Accurate enzyme annotation remains a major bottleneck in translating rapidly growing protein sequence data into biological knowledge. Enzyme Commission (EC) prediction is particularly challenging because enzyme functions are organized hierarchically, annotations are often imbalanced across classes, and sequence similarity alone may be insufficient to resolve functional differences. To address these challenges, we developed ESM-ECForest, a 2-stage framework that combines protein embeddings generated by the pretrained language model ESM-2 (Evolutionary Scale Modeling 2) with random forest classifiers. The first stage distinguishes enzymes from nonenzymes, whereas the second assigns one or more EC numbers to proteins predicted to be enzymatic. On an external benchmark comprising 25,778 protein sequences, ESM-ECForest achieved competitive weighted F1 scores among the evaluated off-the-shelf methods, decreasing from 0.94 at Level 1 to 0.90 at Level 4. The largest relative improvements were observed for lyases (EC 4), ligases (EC 6), and translocases (EC 7), although EC 6 and EC 7 remained the most difficult classes internally. Uniform manifold approximation and projection of the ESM-2 embedding space showed clustering patterns that broadly reflected enzyme functional relationships, suggesting that the pretrained representations capture functionally relevant information. These results support the use of pretrained protein language model embeddings as an effective foundation for enzyme annotation. By combining pretrained protein representations with a lightweight supervised classifier, ESM-ECForest provides a scalable approach for EC prediction and may facilitate functional annotation of proteins from large microbial genomic and metagenomic datasets within the taxonomic and EC-label space represented during model development.

## Introduction

The rapid growth of genome and metagenome sequencing has led to an unprecedented accumulation of protein sequence data. In contrast, experimentally supported functional annotations remain available for only a small fraction of known proteins. This imbalance is particularly evident in microbial datasets, where many predicted proteins show limited similarity to characterized enzymes and therefore remain difficult to annotate reliably. Because enzymes connect gene products to specific biochemical reactions, accurate enzyme annotation plays a central role in interpreting cellular functions and reconstructing metabolic processes [1,2].

The Enzyme Commission (EC) system provides a standardized framework for describing enzyme function through a hierarchical classification scheme that reflects the biochemical reactions catalyzed by enzymes [3–5]. Beyond protein function annotation, EC numbers are widely used in pathway reconstruction, metabolic network analysis, and genome-scale metabolic modeling, where they serve as an important link between protein sequences and biochemical reactions, including their equations and stoichiometry.

Experimental characterization remains the most reliable approach for enzyme annotation [4]. However, functional validation is often constrained by the availability of suitable substrates, cofactors, and assay conditions, making large-scale characterization impractical [2]. As a result, computational approaches have become indispensable for narrowing the gap between sequence availability and functional knowledge. A wide range of computational strategies has been developed for enzyme annotation [6–9], including sequence similarity-based methods [10–12], homology-driven approaches [13,14], structure-informed prediction [15,16], and machine learning (ML) frameworks [17–25]. Sequence alignment tools such as BLAST continue to be widely used because of their simplicity and interpretability. Their effectiveness, however, decreases as sequence similarity declines, particularly when distant homologs possess distinct biochemical functions [26–28]. This challenge has motivated increasing interest in ML approaches that learn sequence–function relationships directly from annotated datasets.

Recent models such as DeepEC [21], ProteInfer [25], and DeepECTransformer [22] have demonstrated that enzyme function can be inferred from amino acid sequences using supervised learning strategies. Nevertheless, the performance of these approaches remains influenced by the characteristics of the available training data. Enzyme annotations are unevenly distributed across EC classes, with well-studied enzyme families represented by substantially more examples than rare or poorly characterized activities [9,29]. Such imbalances complicate model training and can limit predictive performance for underrepresented enzyme classes and newly sequenced proteins [19,21].

Protein language models (PLMs) provide an alternative approach to protein sequence representation. Models such as ESM-2 (Evolutionary Scale Modeling 2) are pretrained on large collections of protein sequences using self-supervised objectives, enabling them to learn contextual representations without relying on functional annotations [29–31]. Previous studies have shown that these representations capture information related to protein structure, evolutionary relationships, and biological function, supporting a variety of downstream applications including structure prediction and functional inference. Unlike conventional supervised approaches, PLMs can exploit information contained in large unlabeled sequence collections, potentially reducing dependence on annotated training datasets [27,32,33]. Despite these advances, the extent to which pretrained protein representations improve enzyme annotation across the EC hierarchy remains unclear [34]. In particular, classes with limited annotation coverage, substantial functional diversity, or low sequence conservation continue to pose challenges for existing prediction methods [17,29,35–38]. Understanding whether information encoded by PLMs can improve robustness in these settings is therefore of considerable practical interest.

In the present study, we developed ESM-ECForest, a 2-stage framework that combines ESM-2-derived protein embeddings with random forest classifiers for enzyme identification and EC prediction. The first stage distinguishes enzymes from nonenzymes, whereas the second performs EC assignment across hierarchy levels for predicted enzymes. By separating sequence representation learning from downstream classification, the framework allows information learned from large-scale protein sequence corpora to be incorporated into enzyme annotation. We evaluated ESM-ECForest using homology-aware data partitions and independent benchmark datasets and compared its performance with established enzyme annotation tools, including DeepEC, ProteInfer, DeepECTransformer, CLEAN, and MMseqs2. The results provide an opportunity to assess the contribution of pretrained protein representations to enzyme annotation and to examine their utility in large-scale functional analysis.

## Methods

### ESM-ECForest: 2-stage identify-then-classify workflow

The ESM-ECForest workflow is summarized in Fig. 1. Protein sequences were first converted into fixed-length numerical representations using ESM-2. The resulting embeddings served as input features for all downstream classifiers. In Stage A, a binary random forest classifier was trained to distinguish enzymes from nonenzymes. Only proteins predicted as enzymes were passed to the EC prediction stage; proteins predicted as nonenzymes were filtered out and received no EC assignment. In Stage B, enzyme sequences are assigned one or more EC numbers through a multilabel classification framework. This 2-stage design separates enzyme detection from functional classification, allowing EC prediction to be restricted to proteins predicted to possess catalytic activity.

**Fig. 1.**
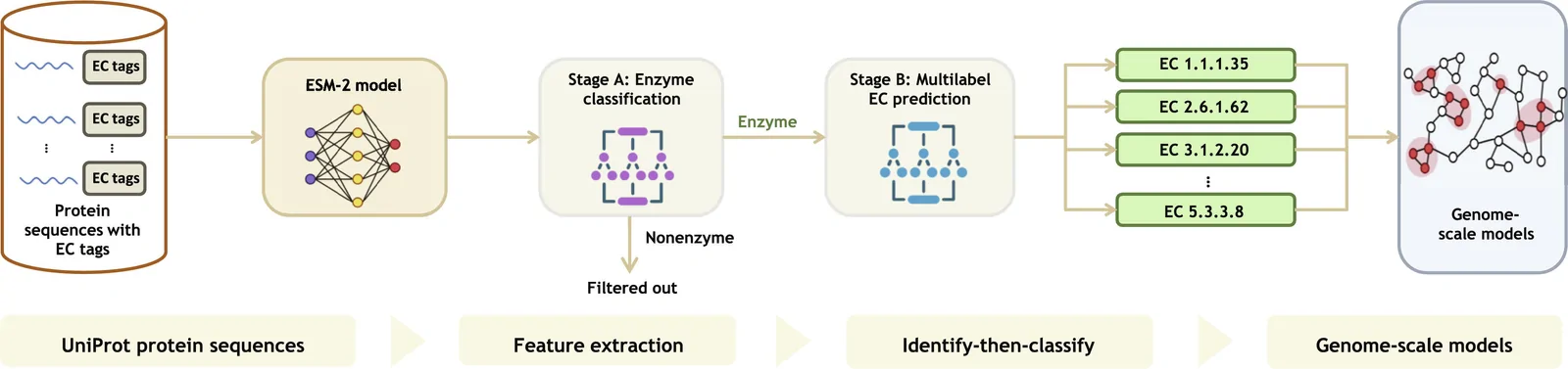
Overview of the ESM-ECForest workflow. Protein sequences obtained from UniProt were transformed into fixed-length embeddings using ESM-2 (Evolutionary Scale Modeling 2). Stage A performed enzyme/nonenzyme classification, and proteins predicted as enzymes were subsequently evaluated by the Stage B classifier for Enzyme Commission (EC) prediction. The resulting EC annotations can be linked to biochemical reactions and used in downstream functional analyses.

Model performance was assessed both across the complete EC hierarchy and within individual top-level EC classes. To facilitate comparison with existing approaches, ESM-ECForest was benchmarked against DeepEC, ProteInfer, DeepECTransformer, CLEAN, and MMseqs2 using an independent evaluation dataset. In addition, uniform manifold approximation and projection (UMAP) was used to visualize the ESM-2 embedding space and to examine the distribution of enzyme and nonenzyme and EC-class representations.

### Dataset construction for ESM-ECForest training and validation

Protein sequences and functional annotations were obtained from UniProtKB/Swiss-Prot (release 2026_01) and used to construct datasets for enzyme identification and EC number prediction. The dataset was assembled from representative prokaryotic and unicellular eukaryotic taxa selected to maximize phylogenetic diversity while limiting the overrepresentation of a small number of model organisms. Inclusion of phylogenetically distant groups was intended to improve taxonomic coverage and support the evaluation of enzyme prediction across diverse regions of protein sequence space. The complete list of taxa included in the study is provided in Table S1.

Data curation followed procedures similar to those adopted in previous enzyme annotation studies [19,23]. Only entries with UniProt annotation scores of 4 or 5 were retained. The annotation score reflects the completeness of sequence annotation and ranges from 1 to 5, with higher values indicating more extensively curated entries [39]. Sequences shorter than 50 amino acids or longer than 5,000 amino acids were excluded. For construction of the original Stage A dataset, sequences were operationally classified as nonenzymes when both catalytic-activity and EC annotations were absent, following criteria used in previous enzyme-prediction studies [23]. Only UniProt entries with annotation scores of 4 or 5 were retained to restrict the dataset to comparatively well-annotated proteins; however, this score reflects annotation completeness and was not interpreted as experimental evidence of nonenzymatic function. We obtained 80,366 proteins classified as nonenzymes. To assess potential label uncertainty in this operational negative class, all 80,366 proteins originally assigned as nonenzymes were subsequently re-evaluated using current UniProt functional annotations. Proteins were categorized as No_enzyme, Uncertain, Likely_enzyme, or Enzyme according to the evidence provided by protein names, family assignments, EC/reaction annotations, catalytic Gene Ontology terms, and annotation cautions. The classification criteria and complete category counts are provided in Table S2. For multifunctional enzymes, all available EC numbers were retained and assigned to the corresponding protein sequence, resulting in a multilabel annotation scheme. Among the 104,648 enzyme sequences in the curated dataset, 21,103 (20.2%) were annotated with multiple EC numbers and were classified as multifunctional enzymes. The final dataset consisted of 104,648 enzymes and 80,366 nonenzymes, corresponding to a total of 185,014 protein sequences.

To reduce sequence redundancy, all sequences were clustered using MMseqs2 (v17-b804f) at 40% sequence identity and 80% coverage. Dataset partitioning was performed at the cluster level with a target train/validation/test ratio of 8:1:1, ensuring that no cluster was shared between subsets. Owing to variation in cluster size, the resulting sequence counts deviated from the target ratio. Stratified sampling was applied whenever possible to preserve class distributions. For enzyme identification, stratification was based on enzyme versus nonenzyme labels, whereas EC classification used the primary EC annotation. Classes represented by fewer than 2 clusters were assigned randomly. Dataset statistics are provided in Table S3.

### Training and validation of ESM-ECForest

Training and model selection were performed using the cluster-based dataset partitions described above. The training set was used for model fitting, the validation set for hyperparameter optimization and threshold selection, and the test set for internal model evaluation during model development.

For Stage A, the binary classifier was trained using enzyme and nonenzyme sequences from the training set. For Stage B, only enzyme sequences were used, with EC annotations encoded as multilabel targets. Multifunctional enzymes contributed to all associated EC labels during training.

Hyperparameters were optimized on the validation set. The evaluated parameters included the number of trees, maximum tree depth, minimum samples required for node splitting, feature-sampling strategy, class-weight configuration, and prediction threshold. The final parameter set was selected according to validation-set performance measured by weighted F1 score.

Class imbalance in the binary enzyme identification task was addressed using balanced class weights. The Stage B one-*vs*-rest random forest classifiers were trained without class weighting. Because terminal EC labels varied substantially in training support, performance was evaluated using macro-, micro-, and support-weighted metrics. Training support for the EC labels is summarized in Table S4; 1,275 labels were represented by a single positive training protein, whereas only 39 had more than 500 positives. After hyperparameter optimization, the final model was fixed and subsequently evaluated using the independent benchmarking dataset described below (“Dataset used for benchmarking” section). The benchmarking dataset was not used for model fitting, parameter tuning, threshold optimization, or model selection. All analyses were performed using a fixed random seed of 42 to ensure reproducibility.

### Protein representation using ESM-2

Protein sequences were encoded using ESM-2, a pretrained PLM trained on large-scale protein sequence data through a masked-token prediction objective [32]. The model generates context-dependent sequence representations from primary amino acid sequences.

In this study, sequence embeddings were generated using the esm2_t6_8M_UR50D model implemented in the FAIR ESM Python package (fair-esm v2.0.0). This model contains 6 transformer layers and approximately 8 million parameters and generates 320-dimensional residue representations. We selected this relatively compact ESM-2 variant deliberately to balance representational capacity with computational accessibility. Because the downstream framework was designed for large-scale enzyme annotation, including genomic and metagenomic applications, using the smallest ESM-2 model substantially reduces the computational cost of embedding generation while retaining the advantages of pretrained contextual protein representations. The objective of the present study was therefore not to identify the optimal PLM, but to assess whether a compact frozen PLM representation could support accurate downstream enzyme classification when combined with conventional ML models.

For each sequence, the beginning-of-sequence (BOS) token representation from the final transformer layer was extracted as a fixed-length feature vector of 320 dimensions. For sequences longer than 1,000 amino acids, nonoverlapping sequence chunks were encoded independently and the resulting embeddings were averaged to obtain a single protein-level representation. These embeddings served as input features for all downstream classification tasks, including enzyme identification and EC prediction [32,38].

### Random forest classifiers

Random forest was used as the downstream classifier for mapping ESM-2 embeddings to enzyme-related labels [40]. All classifiers were implemented in Python using scikit-learn (v1.6.1) and trained on the 320-dimensional ESM-2 embeddings described above. Random forest parameters were determined through preliminary validation experiments. A small number of candidate configurations were evaluated on the validation set, and the final settings were selected based on validation performance and computational efficiency.

In Stage A, enzyme identification was formulated as a binary classification problem. A random forest classifier consisting of 400 trees (maximum depth = 40) was trained to distinguish enzymes from nonenzymes. Class imbalance was addressed using balanced class weights. Additional parameter settings are provided in Table S5.

In Stage B, EC annotation was formulated as a multilabel classification task and was performed only for proteins predicted as enzymes. EC annotations were encoded using MultiLabelBinarizer, and a one-*vs*-rest strategy was adopted in which a separate binary random forest classifier was trained for each EC label. The training dataset contained 3,021 distinct EC labels, resulting in 3,021 individual classifiers. Multiple EC numbers could therefore be assigned to a single protein by predicting more than one positive label. During inference, each Stage B classifier generated a probability score for its corresponding EC label. EC labels with predicted probabilities ≥0.7 were retained as final annotations. The threshold was selected based on validation-set performance and fixed before evaluation on the independent benchmark dataset. A threshold-sensitivity analysis for Stage B showed that a probability threshold of 0.7 yielded the highest macro and weighted F1 scores at EC Level 4 among the tested thresholds (Table S6). Random forest was selected because it can accommodate high-dimensional feature representations without requiring additional feature engineering and has been widely applied to biological classification tasks [41,42]. Its ensemble-based structure also provides a practical approach for handling heterogeneous datasets and nonlinear relationships between protein representations and functional labels.

### Evaluation metrics

Model performance was assessed using precision, recall, and F1 score. True positives (TP), false positives (FP), and false negatives (FN) were determined by comparing predicted labels with reference annotations [9,43].

For a given class:

$$\text{Precision}=\frac{\mathrm{TP}}{\mathrm{TP}+\mathrm{FP}}$$ (1)

$$\text{Recall}=\frac{\mathrm{TP}}{\mathrm{TP}+\mathrm{FN}}$$ (2)

$$F1=\frac{2\times \text{precision}\times \text{recall}}{\text{precision}+\text{recall}}$$ (3)

For the binary enzyme identification task, precision, recall, and F1 score were computed for enzyme versus nonenzyme classification. For EC number prediction, performance was evaluated as multilabel EC prediction across the 4 levels of the EC hierarchy. Each EC annotation associated with a protein was treated as an independent positive label [44]. Missing annotated EC numbers were counted as false negatives, whereas unsupported additional EC predictions were counted as false positives. Predicted and reference EC numbers were compared at EC Levels 1 to 4 after truncation to the corresponding hierarchical level. For example, EC 1.2.3.4 contributed to labels 1, 1.2, 1.2.3, and 1.2.3.4 at Levels 1 to 4, respectively. Duplicate labels generated during truncation were removed prior to metric calculation. Incomplete EC annotations (e.g., 1.2.-.-) were evaluated only at supported hierarchical levels and were excluded from deeper-level comparisons.

Performance was evaluated independently at each EC level using precision, recall, and F1 score. Multilabel EC prediction performance was summarized using macro-, micro-, and support-weighted averages. At each EC level, label-specific precision ($P_{i}$), recall ($R_{i}$), and F1 score ($F1_{i}$) were calculated according to Eqs. (1) to (3), respectively.

For K EC labels, macro-averaged metrics were calculated as

$$P_{\text{macro}}=\left(\frac{1}{K}\right)\sum_{i=1}^{K}P_{i}$$ (4)

$$R_{\text{macro}}=\left(\frac{1}{K}\right)\sum_{i=1}^{K}R_{i}$$ (5)

$$F1_{\text{macro}}=\left(\frac{1}{K}\right)\sum_{i=1}^{K}F1_{i}$$ (6)

Micro-averaged metrics were calculated after pooling true positives, false positives, and false negatives across all labels:

$$P_{\text{micro}}=\frac{\sum_{i}{\mathrm{TP}}_{i}}{\sum_{i}\left({\mathrm{TP}}_{i}+{\mathrm{FP}}_{i}\right)}$$ (7)

$$R_{\text{micro}}=\frac{\sum_{i}{\mathrm{TP}}_{i}}{\sum_{i}\left({\mathrm{TP}}_{i}+{\mathrm{FN}}_{i}\right)}$$ (8)

$$F1_{\text{micro}}=2\frac{P_{\text{micro}}R_{\text{micr}o}}{\left(P_{\text{micro}}+R_{\text{micro}}\right)}$$ (9)

Support-weighted metrics were calculated as

$$M_{\text{weighted}}=\frac{\sum_{i=1}^{K}n_{i}M_{i}}{\sum_{i=1}^{K}n_{i}}$$ (10)

where $M_{i}$ denotes the precision, recall, or F1 score for label i and $n_{i}$ represents the number of reference instances associated with that label.

Confidence intervals (CIs) were estimated by resampling benchmark proteins with replacement for 1,000 replicates. Precision, recall, and F1 score were recalculated for each replicate, and 95% CIs were defined by the 2.5th and 97.5th percentiles of the resulting distributions. This approach captures uncertainty at the protein level but does not account for dependencies among proteins within the same sequence cluster.

Performance was additionally evaluated for each first-level EC class, and class support was reported alongside the corresponding metrics. Proteins without predicted EC annotations were counted as false negatives for the corresponding reference labels. EC classes unsupported by a given method were retained in the evaluation and treated as missing predictions. Undefined precision or recall values were assigned a value of zero. The same evaluation procedure was applied to ESM-ECForest, DeepEC, ProteInfer, DeepECTransformer, CLEAN, and MMseqs2.

### UMAP visualization of the embedding space

The 320-dimensional ESM-2 embeddings were visualized using UMAP [45]. Two-dimensional projections were generated for (a) the complete enzyme/nonenzyme dataset and (b) enzyme sequences colored by first-level EC class. Because the full dataset exceeded 180,000 sequences, a stratified subset of 30,000 proteins was used for visualization. Stratification preserved both enzyme/nonenzyme proportions and first-level EC class distributions. UMAP was implemented using umap-learn (version 0.5.9.post2) with n_neighbors = 15, min_dist = 0.1, metric = “cosine”, and random_state = 42. These projections were used exclusively for qualitative visualization of the embedding space and not as quantitative measures of class separability.

### Dataset used for benchmarking

An independent benchmarking dataset comprising 25,778 protein sequences from 15 prokaryotic and unicellular eukaryotic species was assembled to compare ESM-ECForest with existing enzyme annotation tools. The selected species represent diverse phylogenetic groups and include organisms commonly used in enzyme annotation and metabolic reconstruction studies. The complete species list is provided in Table S7. Among the 14,847 proteins with EC annotations in the benchmarking dataset, 7,528 (50.7%) carried multiple EC numbers and were therefore considered multifunctional enzymes.

Benchmark proteins were not included in the training, validation, or internal test sets used for the development of ESM-ECForest. Sequence similarity between benchmark and training proteins was assessed using MMseqs2. The highest observed sequence identity was 38.4%, which remained below the 40% sequence identity threshold used for MMseqs2 clustering during dataset construction. This analysis therefore controls overlap with the downstream supervised training data used to fit ESM-ECForest. However, because ESM-2 was pretrained independently on a large protein sequence corpus, we cannot exclude the possibility that benchmark proteins or related sequences were encountered during ESM-2 pretraining.

### Benchmarking against existing tools

ESM-ECForest was benchmarked against 5 sequence-based enzyme annotation tools: DeepEC, ProteInfer, DeepECTransformer, CLEAN, and MMseqs2 [12,17,21,22,25]. These methods were selected because they represent different modeling strategies for protein function prediction, including convolutional neural networks (CNNs), dilated convolutional architectures, and transformer-based approaches.

DeepEC performs EC number prediction using CNNs supplemented by homology-based annotation for sequences that cannot be confidently classified by the neural network component [21]. ProteInfer predicts enzyme functions directly from unaligned amino acid sequences using a deep dilated convolutional architecture [25]. DeepECTransformer combines transformer-based sequence modeling with homology search and was developed for large-scale enzyme annotation, particularly in microbial genomes [22]. CLEAN uses PLM embeddings together with contrastive learning for EC prediction [17]. MMseqs2 was included as a nearest-neighbor EC transfer baseline, assigning EC annotations based on the closest match in the training set [12].

Benchmarking was performed using an external dataset described in the “Dataset used for benchmarking” section. Official implementations provided by the respective authors were used for all methods. Unless otherwise stated, analyses were performed using the recommended default settings. Repository commit hashes are reported in Table S8 where formal software releases were unavailable. All predictions were mapped to a common EC number representation before evaluation.

Because the evaluated methods differ in EC coverage and output format, all predictions were converted to a common EC representation before evaluation. Precision, recall, weighted F1 score, and per-class F1 score were calculated as described in the “Evaluation metrics” section. Incomplete EC annotations were evaluated only at the highest supported EC level. Unsupported classes were retained in the overall hierarchical evaluation as missing predictions, whereas unsupported class-specific values were displayed as N/A in per-class plots.

Runtime and peak memory usage were evaluated using the benchmark dataset described above. Wall-clock time and peak memory consumption were measured during prediction only, from execution of the prediction command to completion. Preprocessing steps unrelated to prediction, including software installation, database and model downloads, and output standardization, were excluded. Hardware and software specifications of the benchmarking environment are summarized in Table S9.

## Results

### Performances of ESM-ECForest

#### Enzyme identification performance of Stage A

Stage A of ESM-ECForest was designed to distinguish enzymes from nonenzymes prior to EC annotation. On the independent benchmarking dataset (*n* = 25,778 proteins), the model achieved a precision of 0.975 (95% CI, 0.972 to 0.977), a recall of 0.977 (95% CI, 0.975 to 0.980), and an F1 score of 0.976 (95% CI, 0.974 to 0.978) (Fig. 2A). The complete performance results are provided in Table S10.

**Fig. 2.**
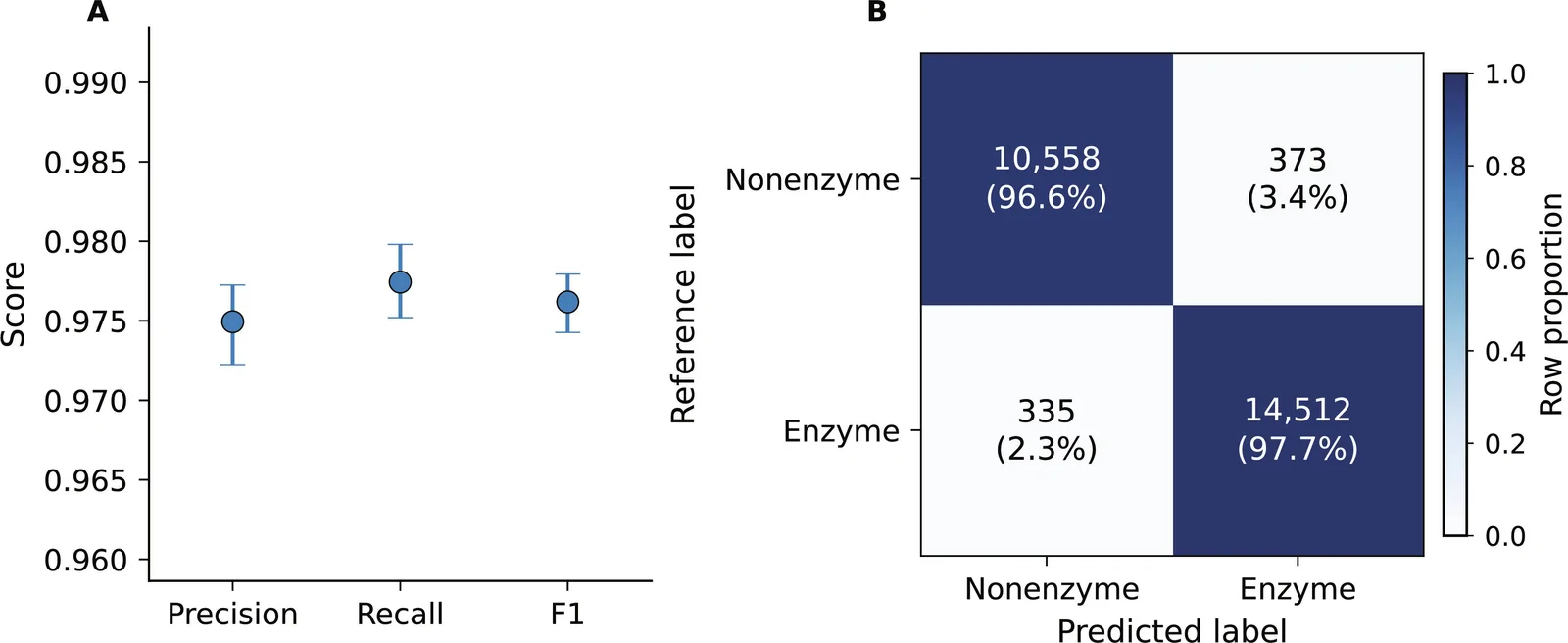
Binary enzyme identification performance of ESM-ECForest. (A) Precision, recall, and F1 score evaluated on the independent benchmarking dataset. Error bars denote bootstrap 95% confidence intervals. (B) Confusion matrix for enzyme and nonenzyme classification. Values indicate the number of proteins, with row-wise percentages shown in parentheses.

The corresponding confusion matrix revealed low error rates for both classes (Fig. 2B), with 373 nonenzymes incorrectly classified as enzymes and 335 enzymes classified as nonenzymes. As a result, 97.7% of enzymes and 96.6% of nonenzymes were correctly identified. Together, these findings indicate that Stage A achieves robust and well-balanced binary classification performance, providing a reliable foundation for subsequent EC number prediction.

Because the absence of an enzyme annotation does not necessarily constitute definitive evidence of nonenzymatic function, we additionally re-evaluated the 80,366 proteins originally assigned to the nonenzyme class using current UniProt functional annotations. The reassessment used protein names and family assignments together with available EC numbers, catalytic-activity/reaction annotations, catalytic Gene Ontology terms, and UniProt annotation qualifiers or caution statements. An ordered rule-based classification was applied to distinguish evidence of catalytic activity from noncatalytic proteins, including inhibitors, structural or regulatory proteins, inactive homologs/pseudoenzymes, and noncatalytic subunits of enzyme complexes. Proteins with annotated catalytic reaction or catalytic GO annotation were classified as “Enzyme”. Proteins whose name, family assignment, or tentative functional annotation strongly suggested catalytic activity, but for which explicit catalytic annotation was lacking or insufficient, were classified as “Likely_enzyme”. Proteins with a recognized noncatalytic function or an explicitly noncatalytic subunit role and no evidence of catalytic activity were classified as “No_enzyme”. Cases with insufficient, ambiguous, or conflicting functional evidence were classified as “Uncertain”. For UniProt accessions that were no longer available in the current release, annotations were recovered where possible through exact-sequence matches to archived UniParc entries. Among the protein sequences, 49,396 (61.5%) had current functional annotations supporting a noncatalytic role, whereas 18,120 (22.5%) remained uncertain. A further 8,418 (10.5%) showed protein-name, family, or tentative catalytic evidence suggesting possible enzyme activity, and 4,432 (5.5%) had current EC, reaction, or catalytic GO annotations supporting enzyme activity (Table S11). Stage A prediction probabilities differed across these categories, with the lowest mean enzyme probability observed for the No_enzyme group and the highest for the Enzyme group (Table S2 and “Enzyme identification performance of Stage A” section).

#### EC prediction performance of the complete ESM-ECForest framework

Following the evaluation of binary enzyme identification, we assessed the end-to-end performance of ESM-ECForest for multilabel EC prediction evaluated across Levels 1 to 4 of the EC hierarchy (Fig. 3). As expected, predictive performance gradually decreased with increasing annotation depth under all averaging schemes (Fig. 3A to C). The decline was most pronounced for macro-averaged metrics, whereas micro- and weighted-average metrics remained comparatively stable across hierarchy levels.

**Fig. 3.**
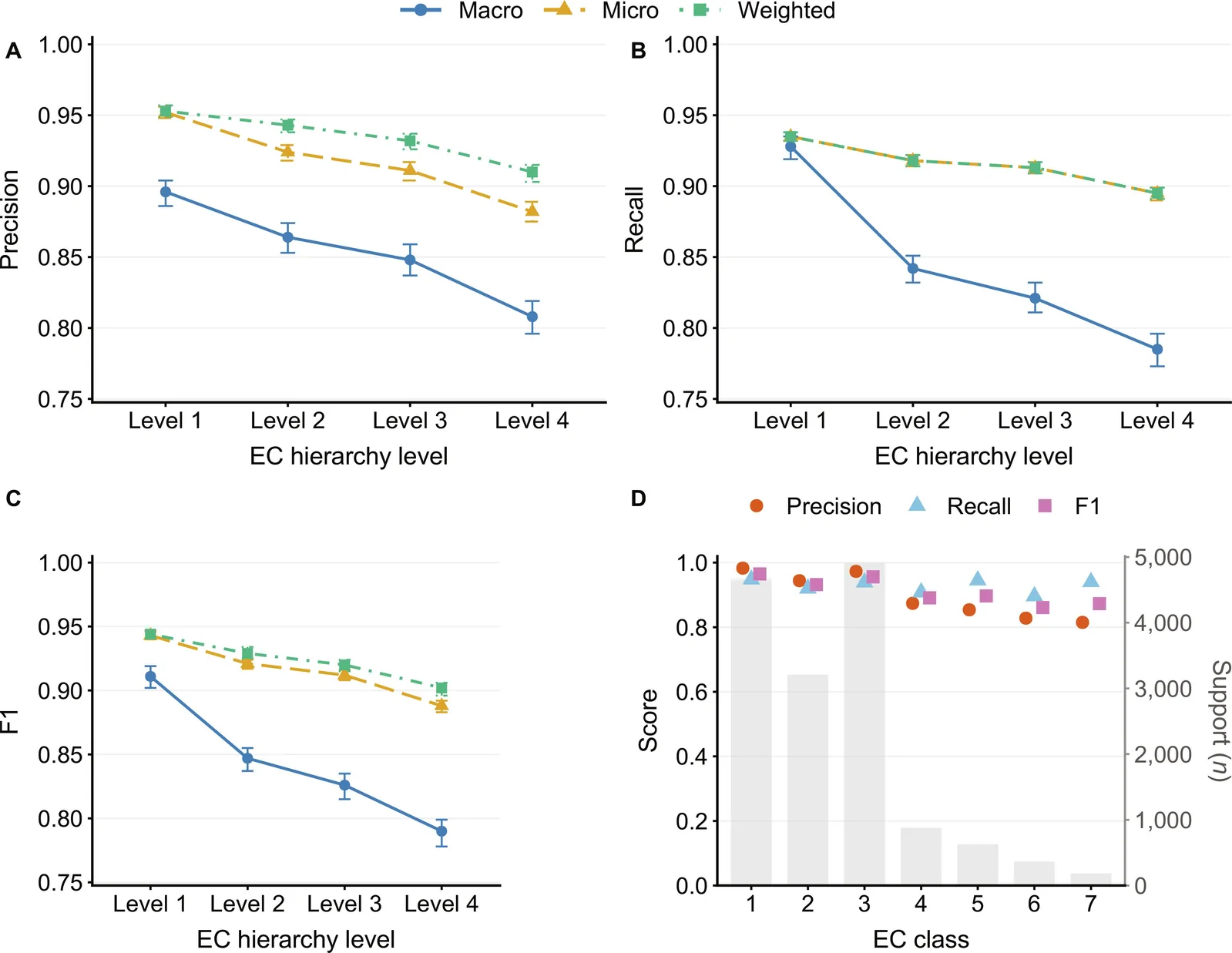
Multilabel Enzyme Commission (EC) prediction performance across EC hierarchy levels and first-digit EC classes of ESM-ECForest. (A to C) Precision, recall, and F1 score across EC hierarchy Levels 1 to 4 using macro-, micro-, and support-weighted averaging. Points indicate performance estimates on the independent benchmarking dataset, and error bars denote 95% confidence intervals obtained from 1,000 bootstrap resamples. (D) Precision, recall, and F1 score for individual first-digit EC classes at Level 1. Gray bars indicate the number of reference annotations for each class (support; right *y*-axis).

At EC Level 1, macro-, micro-, and weighted-average F1 scores were 0.911 (95% CI, 0.902 to 0.919), 0.943 (95% CI, 0.940 to 0.946), and 0.944 (95% CI, 0.941 to 0.947), respectively. At Level 4, these values decreased to 0.790 (95% CI, 0.778 to 0.799), 0.888 (95% CI, 0.883 to 0.892), and 0.902 (95% CI, 0.896 to 0.906). Similar trends were observed for precision and recall (Fig. 3A and B). Macro precision decreased from 0.896 to 0.808, while macro recall decreased from 0.928 to 0.785 between Levels 1 and 4. In contrast, weighted-average metrics showed only modest declines across hierarchy levels and remained substantially higher than the corresponding macro-averaged metrics. Detailed performance results are provided in Table S12A. Protein-level multilabel evaluation at EC Level 4 yielded a sample-based F1 score of 0.886 and an exact-match accuracy of 0.844. Proteins annotated with a single EC number achieved a higher sample-based F1 score than multifunctional proteins (0.963 versus 0.806; Table S13).

To further examine class-specific behavior, performance was evaluated separately for each first-level EC category (Fig. 3D). EC 1 to 3 achieved F1 scores of 0.965, 0.932, and 0.956, respectively, whereas EC 4 to 7 showed lower performance, with F1 scores ranging from 0.861 to 0.897. Precision and recall also varied across EC classes, indicating differences in classification difficulty among enzyme categories. Detailed class-specific results are provided in Table S12B. Despite these differences, all first-level EC classes achieved F1 scores above 0.86, demonstrating robust prediction performance across the major enzyme categories. Per-EC F1 scores were compared with the log-transformed number of training proteins associated with each EC label (Fig. S1). Performance varied widely across EC labels, particularly among low-frequency labels, and no clear relationship between training-set frequency and per-EC performance was observed. Proteins were also grouped according to their maximum sequence identity to the training dataset. At EC Level 4, both macro- and weighted-average F1 scores increased with sequence identity (Fig. S2 and Table S14). Nonzero performance was observed across all sequence-identity ranges, including proteins without detectable matches in the training dataset.

Because proteins longer than 1,000 amino acids were represented using averaged embeddings across nonoverlapping sequence chunks, we examined performance by protein length. At EC Level 4, proteins >1,000 amino acids showed lower performance than proteins ≤1,000 amino acids, with macro F1 decreasing from 0.792 to 0.724 and weighted F1 from 0.905 to 0.863. More details are summarized in Table S15A to D.

To evaluate the contribution of individual components, we performed ablation analyses of the gating strategy, embedding representation, and downstream classifier. Using the true enzyme status instead of the predicted Stage A gate increased EC Level 4 macro and weighted F1 from 0.790 to 0.858 and from 0.902 to 0.921, respectively. Removing the Stage A gate and applying Stage B directly reduced weighted F1 to 0.863. Mean residue pooling achieved a higher weighted F1 than BOS pooling (0.916 *vs* 0.902), whereas BOS pooling resulted in a slightly higher macro F1 (0.790 *vs.* 0.784). Random forest and gradient boosting machine achieved similar weighted F1 scores (0.902 and 0.901), although random forest yielded a higher macro F1 (0.790 *vs* 0.736). Logistic regression performed substantially worse than both tree-based classifiers. Full results are shown in Table S16.

#### UMAP visualization of the ESM-2 embedding space

To examine the organization of the pretrained protein representations, the 320-dimensional ESM-2 embeddings were projected into 2 dimensions using UMAP (Fig. 4). The projection containing both enzyme and nonenzyme sequences revealed partial separation between the 2 groups (Fig. 4A). Several regions were enriched for either enzymes or nonenzymes, although overlap between the 2 populations was also observed.

**Fig. 4.**
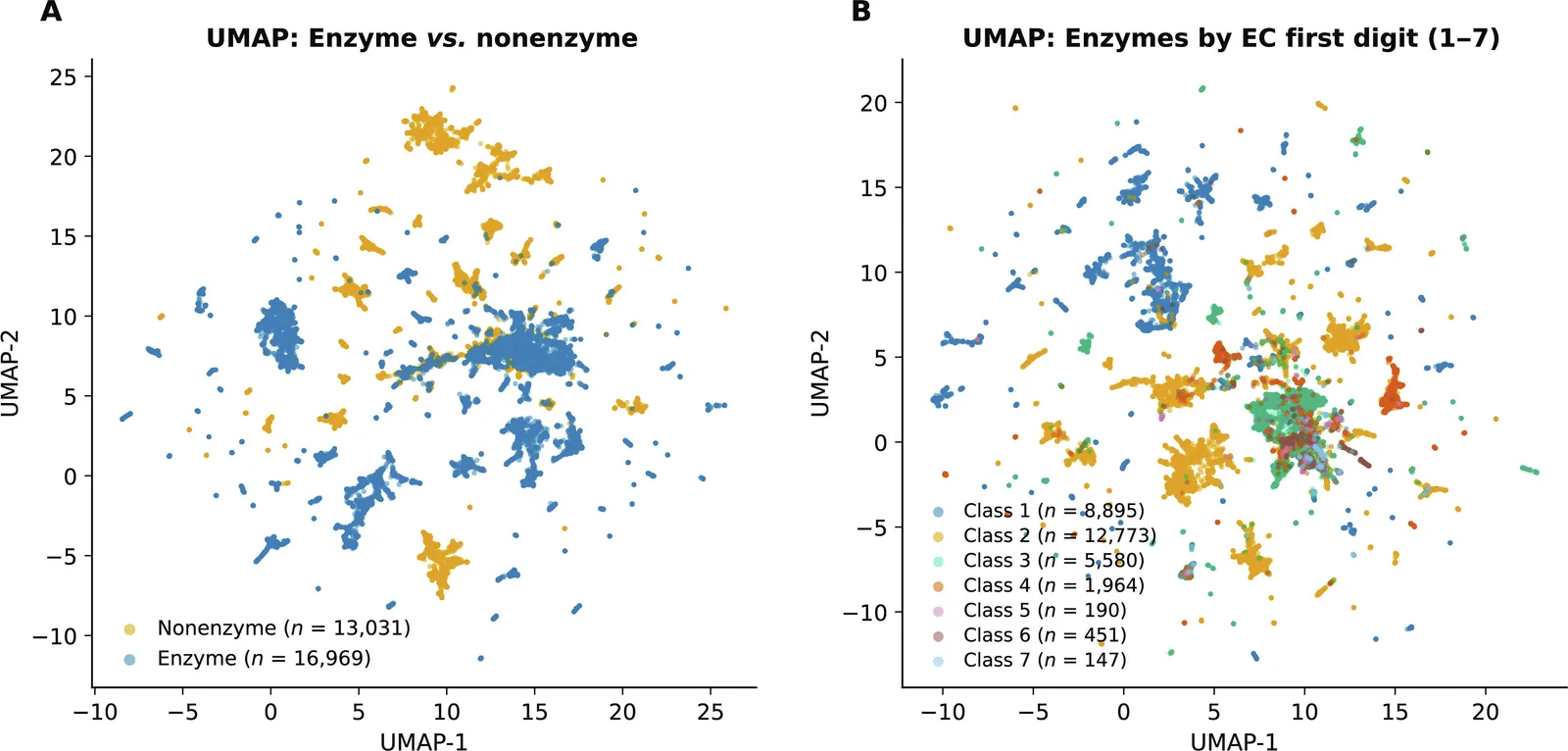
Uniform manifold approximation and projection (UMAP) visualization of ESM-2 (Evolutionary Scale Modeling 2) protein embeddings. (A) Projection of enzyme and nonenzyme sequences. (B) Projection of enzyme sequences colored according to first-level Enzyme Commission (EC) class.

A second UMAP projection was generated using enzyme sequences only and colored according to first-level EC class (Fig. 4B). Distinct enrichment patterns were observed for several EC categories. The largest classes, including EC 1, EC 2, and EC 3, occupied broader regions of the embedding space, whereas EC 5, EC 6, and EC 7 were represented by smaller and more dispersed clusters. Considerable overlap remained among EC classes, particularly in regions containing multiple functional groups.

The UMAP projections indicate that proteins with different annotations are not uniformly distributed within the ESM-2 embedding space. Similarities in the distribution of proteins sharing common functional labels were observed for both enzyme/nonenzyme classification and first-level EC categories. These patterns provide a qualitative overview of the organization of the projected embeddings but do not establish the degree of class separability in the original 320-dimensional space.

### Benchmarking against existing EC enzyme-prediction tools

To assess the performance of ESM-ECForest, we compared it with 5 representative sequence-based EC prediction methods: DeepEC, ProteInfer, DeepECTransformer, CLEAN, and MMseqs2 nearest-neighbor EC transfer. Together, these methods cover CNN-, dilated-convolutional-network-, and transformer-based architectures that are widely used for enzyme function prediction.

#### Overall benchmark performance

We compared ESM-ECForest with DeepECTransformer, ProteInfer, DeepEC, CLEAN, and MMseqs2 nearest-neighbor transfer across the 4 levels of the EC hierarchy (Fig. 5). All tools were evaluated using their official implementations and default settings. Because the methods differ in training datasets, annotation releases, supported EC vocabularies, thresholds, and prediction coverage, the comparison reflects the performance of the released tools rather than architecture alone. Full results are provided in Table S17. A common-label analysis restricted to EC classes supported by all methods (EC 1 to EC 6) is reported in Table S18. Performance decreased with increasing EC hierarchy depth for all methods (Fig. 5A and B). ESM-ECForest achieved weighted F1 scores of 0.944, 0.929, 0.920, and 0.902 at EC Levels 1 to 4, respectively, outperforming DeepECTransformer, ProteInfer, DeepEC, and MMseqs2 across all levels. CLEAN achieved weighted F1 scores of 0.951, 0.934, 0.916, and 0.907 and showed comparable overall performance. At EC Level 4, CLEAN reached a slightly higher weighted F1 than ESM-ECForest (0.907 *vs* 0.902), whereas ESM-ECForest achieved a slightly higher macro F1 (0.790 *vs.* 0.786). MMseqs2 performed substantially worse at Level 4, with weighted and macro F1 scores of 0.503 and 0.301, respectively.

**Fig. 5.**
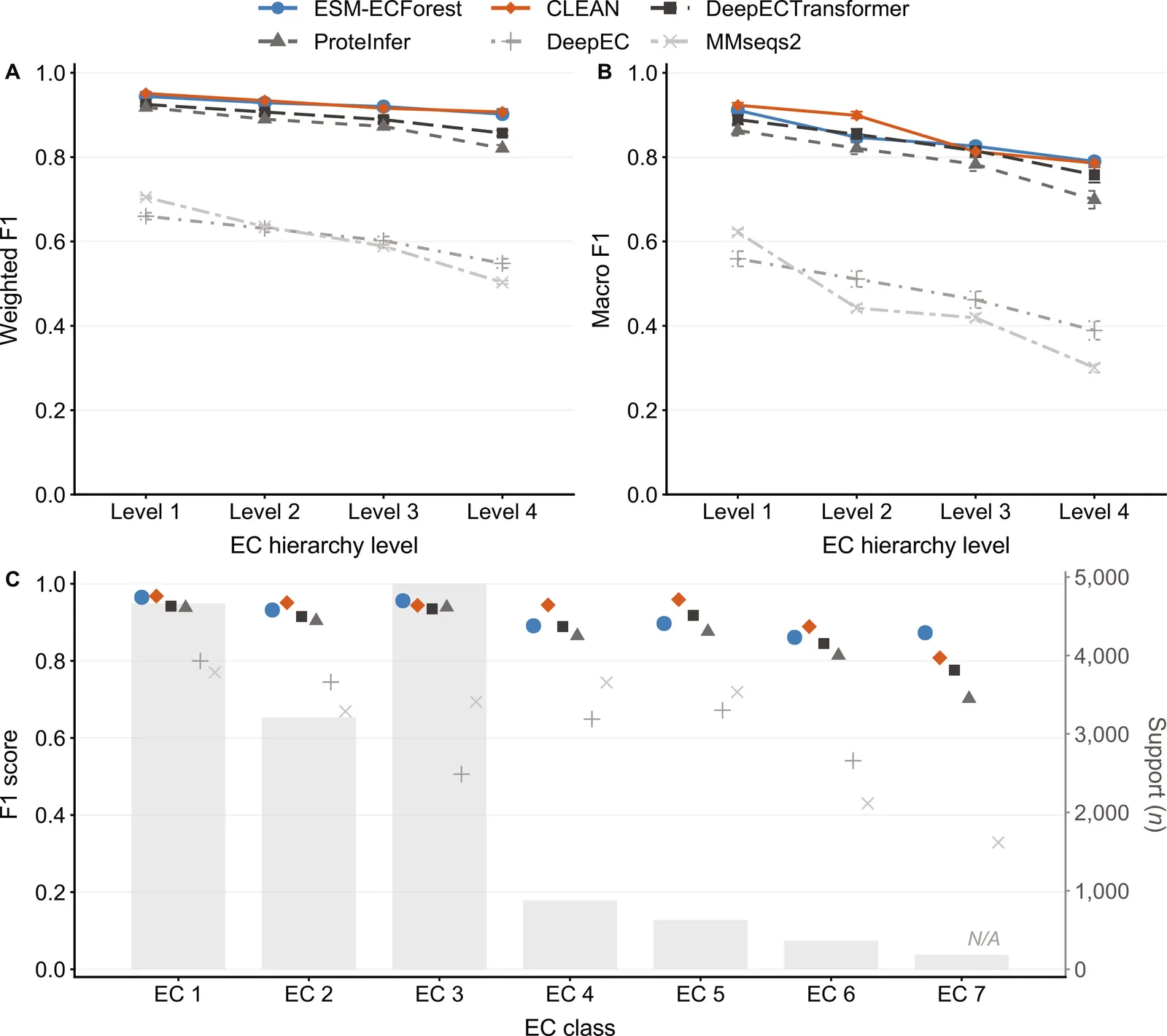
Benchmarking comparison of ESM-ECForest and existing enzyme annotation tools. (A) Weighted F1 scores across Enzyme Commission (EC) Levels 1 to 4. (B) Macro F1 scores across EC Levels 1 to 4. (C) Class-wise F1 scores at EC Level 1. Gray bars indicate the number of proteins in each EC class (right *y*-axis). Error bars denote 95% bootstrap confidence intervals. MMseqs2 represents nearest-neighbor EC transfer using the ESM-ECForest training set. EC class 7 is not supported by the original DeepEC implementation and is shown as N/A. Exact numerical values underlying all panels are provided in Table S17.

Similar trends were observed for macro F1 (Fig. 5B). ESM-ECForest achieved macro F1 scores of 0.911, 0.847, 0.826, and 0.790 from Levels 1 to 4. CLEAN was higher at Levels 1 and 2, whereas ESM-ECForest was higher at Levels 3 and 4. All remaining methods produced lower macro F1 scores than ESM-ECForest at Level 4. Class-wise analysis at EC Level 1 showed stable performance across enzyme classes (Fig. 5C). ESM-ECForest achieved F1 scores of 0.965, 0.932, and 0.956 for EC classes 1 to 3 and 0.861 to 0.897 for EC classes 4 to 7. CLEAN showed similar class-level performance across the 7 top-level EC classes. The largest difference relative to the earlier baselines was observed for EC class 7, where ESM-ECForest achieved an F1 score of 0.873, compared with 0.776 for DeepECTransformer and 0.702 for ProteInfer. EC class 7 predictions were not available from DeepEC.

#### Runtime and memory comparison

Runtime and peak memory usage were evaluated on the benchmark FASTA dataset using 24 central processing unit (CPU) threads and no graphics processing unit (GPU) acceleration. MMseqs2 nearest-neighbor transfer was the fastest method, completing prediction in 1.3 min with a peak memory usage of 0.19 GB. DeepECTransformer required 6.4 min and 9.1 GB of random-access memory (RAM), followed by DeepEC (8.6 min, 18.9 GB). ESM-ECForest completed prediction in 11.6 min with 13.4 GB of RAM, whereas ProteInfer required 44.2 min and 36.4 GB of RAM. CLEAN required the longest runtime under our local CPU-only setup (866 min, 9.83 GB RAM) (Table 1).

**Table 1.** Runtime and memory usage of enzyme annotation tools. Runtime was measured on the benchmark FASTA dataset using 24 CPU threads without GPU acceleration. Peak RAM denotes maximum memory usage during prediction. MMseqs2 runtime excludes database construction and indexing. ProteInfer was evaluated with its default workflow, including Pfam and Gene Ontology annotation. CLEAN was evaluated using a local CPU-only workflow.

Tool	CPU threads	Peak RAM (GB)	Runtime (min)
DeepECTransformer	24	9.1	6.4
DeepEC	24	18.9	8.6
ESM-ECForest	24	13.4	11.6
ProteInfer	24	36.4	44.2
MMseqs2	24	0.19	1.3
CLEAN	24	9.83	866

CPU, central processing unit; GPU, graphics processing unit; RAM, random-access memory

ESM-ECForest required more computation than MMseqs2, DeepECTransformer, and DeepEC, but less than ProteInfer and CLEAN under this CPU-only workflow. Runtime and memory measurements depend on the tested software implementations and execution settings. Because all methods were evaluated without GPU acceleration, the reported runtimes reflect performance under the benchmark conditions used in this study. Runtime may differ substantially on GPU hardware, particularly for deep-learning methods optimized for accelerator-based inference. Although ESM-ECForest uses nonneural downstream classifiers, Stage B comprises 3,021 one-*vs*-rest random forest models. ESM-ECForest combines frozen PLM embeddings with nonneural downstream classifiers. ProteInfer was evaluated using its default workflow, which additionally produces Pfam and Gene Ontology annotations; therefore, the reported runtimes reflect end-to-end workflow performance rather than EC prediction alone.

## Discussion

The rapid expansion of protein sequence databases continues to outpace the experimental characterization of enzyme functions, creating a persistent gap between sequence availability and functional annotation. Our results indicate that pretrained PLM embeddings, combined with a 2-stage prediction framework, can effectively support multilabel EC prediction across EC hierarchy levels. The performance gains observed across multiple EC levels suggest that language-model-derived representations encode biologically meaningful features relevant to enzyme function. Nevertheless, the model does not perform uniformly across all EC categories, and reduced accuracy in certain classes points to intrinsic challenges associated with functional heterogeneity and data scarcity. These findings provide insight into both the strengths and current limitations of embedding-based enzyme annotation approaches and are relevant for applications that depend on accurate enzyme function prediction, including genome-scale metabolic reconstruction.

### PLM embeddings enable robust EC prediction across hierarchy levels

Our results show that strong EC prediction performance across hierarchy levels can be achieved using pretrained sequence representations combined with a relatively lightweight downstream classifier. Although ESM-ECForest uses a relatively simple random forest classifier, its performance remained stable across successive EC levels, with only a moderate decrease as the prediction task moved toward more specific functional assignments. The limited performance decline suggests that information relevant to both broad and fine-grained enzyme functions is retained within the ESM-2 embedding space.

This interpretation is also reflected in the UMAP projection. Enzyme and nonenzyme sequences were distributed in partially distinct regions, while enzymes belonging to the same first-level EC category showed a tendency to cluster together. Complete separation would not be expected given the considerable functional and evolutionary overlap among enzyme families. Nevertheless, the observed structure indicates that functionally related proteins occupy similar regions of the embedding space before any task-specific training is performed [32,33,46]. However, these patterns should be interpreted cautiously, as dimensionality reduction can introduce or accentuate apparent clustering. Consequently, the UMAP analysis does not, in itself, establish quantitative class separability in the original embedding space or demonstrate that the representations explicitly encode catalytic mechanisms or structural determinants.

An important component of the framework is the initial discrimination between enzymes and nonenzymes. Stage A achieved an F1 score of 0.976 on the independent benchmark, with similarly high precision and recall, indicating that ESM-2 representations contain sufficient information to distinguish catalytic from noncatalytic proteins even under the sequence-redundancy-controlled evaluation used here. This initial filtering step is particularly relevant for end-to-end annotation because false-negative predictions at Stage A cannot subsequently receive an EC assignment, whereas false-positive predictions are passed to Stage B and may generate inappropriate EC annotations. The low error rate observed at Stage A therefore limits the propagation of classification errors into the downstream EC-prediction step and supports the use of an identify-then-classify architecture.

The model showed relatively consistent behavior across different EC classes, including EC 4, EC 6, and EC 7, which are frequently associated with greater sequence heterogeneity and comparatively limited annotation resources [17]. Performance in these categories may reflect the ability of pretrained embeddings to capture properties that are not restricted to individual enzyme families. Instead of depending primarily on family-specific sequence patterns, the representation appears to preserve broader functional signals that remain informative across diverse enzyme groups [29].

This observation is unlikely to be specific to the present model and may instead reflect properties of PLM representations that have been reported previously [38]. Representations generated by ESM-2 have been associated with structural, evolutionary, and functional characteristics of proteins, despite being trained without explicit functional supervision [32]. The success of ESMFold, in line with earlier studies on unsupervised protein language modeling [33,47,48], further illustrated the extent to which information embedded in PLM representations can learn representations reflecting biological structure and function and support downstream biological inference. In the context of enzyme annotation, the present results suggest that these embeddings contain sequence-level constraints related to catalytic function, domain architecture, and protein structure, providing a useful basis for hierarchical EC prediction [34].

### Why ESM embeddings help

A notable characteristic of ESM-ECForest is its separation of protein representation learning from supervised enzyme classification. Rather than deriving sequence features directly from EC annotations, the framework first generates protein embeddings using the pretrained ESM-2 encoder and then applies random forest classifiers to the resulting representations. In this way, learning the general properties of protein sequences and learning function-specific decision boundaries are treated as 2 related but distinct tasks.

One possible explanation for the effectiveness of ESM-derived representations lies in the pretraining objective. ESM-2 is trained to recover masked amino acids from their surrounding sequence context:

$$L_{\mathrm{MLM}}=-\sum_{i\in M}\log{\;p}_{\theta }\left(a_{i}|x_{\setminus M}\right)$$ (11)

where $L_{\mathrm{MLM}}$ is the masked language modeling loss used to train ESM-2, $x=\left(a_{1},a_{2},\ldots ,a_{L}\right)$ denotes an amino acid sequence of length L, M denotes the set of masked positions, $a_{i}$ is the true amino acid at position i, and $p_{\theta }\left(a_{i}|x_{\setminus M}\right)$ is the probability that the model assigns to the correct amino acid $a_{i}$ given the surrounding sequence. Optimizing this objective requires the model to integrate information across multiple sequence positions rather than relying only on local patterns. As a result, the learned representations can capture constraints associated with protein structure, evolutionary conservation, and biochemical function [33,34]. Self-attention may further allow information from distant positions in the primary sequence to contribute to the representation, which is relevant because catalytic residues can be separated in sequence while becoming spatially adjacent in the folded protein.

In ESM-ECForest, the final-layer BOS token representation was used as a fixed-length sequence-level embedding, with chunk-level embeddings averaged only for sequences longer than the ESM-2 input limit. Random forest classifiers were then trained on these embeddings to distinguish enzymes from nonenzymes and to assign EC labels. Conceptually, the 2-stage prediction procedure can be written as

$$P\left(EC_{i}|x\right)\approx P\left(E=1|z\right)\times P\left(EC_{i}|E=1,z\right)$$ (12)

where E=1 denotes the event that a protein is enzymatic and *z* denotes the fixed-length ESM-2 sequence embedding. The first stage estimates whether a sequence is likely to encode an enzyme, whereas the second stage assigns EC numbers only to sequences classified as enzymatic. This 2-stage framework separates enzyme identification from EC classification and limits EC assignment to proteins predicted as enzymes.

### Benchmarking reveals competitive performance among existing sequence-based tools

The evaluated tools span several sequence-based annotation strategies, including CNNs, transformer models, contrastive learning, and sequence-similarity transfer. These approaches differ primarily in how sequence information is represented and used for prediction.

These approaches differ mainly in how sequence context is represented and incorporated into prediction. CNN-based methods such as DeepEC [19,38] and ProteInfer [49] rely on local sequence patterns and motif detection. ProteInfer extends the receptive field through dilated convolutions, enabling information from more distant sequence positions to be incorporated during prediction. In contrast, transformer-based models use attention mechanisms to capture relationships between residues regardless of sequence distance, a property that may be particularly relevant for enzyme function prediction [22,50].

ESM-ECForest differs from several of the compared methods in that protein representations are learned before EC classification. Protein embeddings are generated by a transformer model pretrained on large-scale protein sequence datasets and subsequently used for downstream prediction. However, the observed benchmark differences cannot be attributed to model architecture alone, as the evaluated methods also differ in training data, EC coverage, and implementation. Within this setting, ESM-ECForest generally outperformed DeepEC, ProteInfer, DeepECTransformer, and MMseqs2 nearest-neighbor transfer, while CLEAN achieved comparable performance. Overall, ESM-ECForest ranked among the best-performing methods evaluated in this study.

The advantage of ESM-ECForest was most evident for EC classes 4, 6, and 7, which contain diverse functions and relatively limited annotation resources [4,17,34]. In such cases, pretrained embeddings may capture functional relationships beyond direct sequence similarity [32,33], allowing the downstream classifier to separate EC classes more effectively.

Computational requirements also varied across methods. MMseqs2 was the fastest method, whereas DeepECTransformer and DeepEC required less computation than ESM-ECForest. For ESM-ECForest, most computational cost originated from ESM-2 embedding generation. CLEAN achieved similar predictive performance but required a substantially longer runtime under the evaluated CPU-only workflow. The benchmark should be interpreted as a comparison of released tools rather than a controlled comparison of model architectures. Differences in training datasets, EC coverage, annotation procedures, confidence thresholds, and auxiliary prediction modules may all influence performance. In addition, because complete training datasets or accession lists were not available for all competing tools, potential overlap between the benchmark dataset and competitor training data could not be assessed for every method. DeepEC, for example, does not support EC class 7. Within these evaluation settings, ESM-ECForest combined competitive predictive performance with moderate computational requirements for multilabel EC annotation across hierarchy levels.

### Biochemical complexity limits prediction in EC 6 and EC 7

Despite the overall improvement achieved by ESM-ECForest, EC 6 and EC 7 remained among the most difficult categories to predict accurately. This trend is unlikely to arise solely from the limitations of the prediction model and may instead reflect intrinsic differences in the biological characteristics of these enzyme classes. Ligases (EC 6) catalyze bond-forming reactions that are typically coupled to energy consumption, whereas translocases (EC 7) mediate the movement of ions or molecules across membranes or their separation within membrane systems. In addition to the diversity of transported substrates, EC 7 enzymes are further subdivided according to the energetic mechanisms that drive transport [51].

A major challenge is the substantial functional heterogeneity present within both classes. Enzymes assigned to the same top-level EC category may differ considerably in substrate specificity, catalytic mechanism, cofactor requirements, and domain organization. Consequently, proteins sharing an EC 6 or EC 7 designation do not necessarily exhibit strong sequence similarity, making sequence-based discrimination inherently more difficult than for enzyme groups with more conserved functional architectures.

The challenge is particularly apparent for translocases. Many EC 7 enzymes are membrane-associated proteins or components of multisubunit complexes whose activities depend on factors extending beyond the primary amino acid sequence. Transmembrane topology, oligomeric state, cellular localization, electrochemical gradients, and coupling to adenosine triphosphate hydrolysis or redox processes can all contribute to biological function. Although some of this information is reflected indirectly in sequence patterns, it may not be fully captured by sequence embeddings alone, especially when different transport systems employ distinct structural solutions to achieve similar functions [34,52].

Dataset characteristics may further contribute to the observed performance differences. Compared with oxidoreductases, transferases, or hydrolases, EC 6 and EC 7 enzymes are represented by fewer experimentally characterized examples in many public annotation resources [17,53]. EC 6 and EC 7 were represented by 551 proteins in our benchmarking dataset, compared with 14,296 for the more abundant EC classes (EC 1 to 5). Reduced annotation density limits the diversity of functional patterns available during model training and increases the influence of incomplete or uncertain reference labels. Under such conditions, benchmark performance may reflect not only prediction errors but also the limitations of the available annotations themselves.

Taken together, the residual errors observed for EC 6 and EC 7 are consistent with the underlying biochemical complexity of these enzyme classes. Further improvements will likely require the integration of information beyond primary sequence, including structural features, membrane topology, active-site and ligand-binding information, domain composition, protein-complex organization, and reaction-level biochemical constraints [34]. Such information may help resolve functional distinctions that are difficult to infer from sequence data alone.

### Implications for metabolic annotation and genome-scale modeling

The main practical value of EC prediction is that it links protein sequences to biochemical reactions. EC numbers provide a functional bridge from gene products to reaction-level annotations, which form the basis of gene–protein–reaction associations in genome-scale metabolic models (GEMs) [54–56]. GEMs represent organism-wide metabolic networks and can be used to simulate metabolic fluxes under different genetic or environmental conditions. By connecting genes to metabolic reactions, these annotations enable the systematic reconstruction and analysis of cellular metabolic networks.

In microbial systems, improved enzyme annotation can facilitate several stages of GEM development [55]. One immediate application is the functional characterization of proteins that remain incompletely annotated. Predicted EC assignments may also assist in identifying candidate genes associated with missing metabolic reactions during gap-filling procedures [57]. In addition, improved enzyme annotations can support the refinement of existing gene–protein–reaction relationships by providing evidence for alternative or previously unrecognized enzyme candidates. Such applications are particularly relevant for *Saccharomyces cerevisiae*, where community-curated reconstructions such as Yeast9 and yeast-GEM continue to serve as important resources for metabolic engineering, and multi-omics integration [55,58,59].

At the same time, the connection between EC annotation and cellular metabolism should not be overstated. An EC prediction reflects the potential catalytic activity encoded by a protein sequence rather than its realized biological function under a specific condition. Whether an enzyme contributes to metabolic flux *in vivo* depends on additional factors, including gene expression, protein abundance, subcellular localization, substrate availability, regulatory mechanisms, and environmental context. Consequently, EC predictions are most informative when considered alongside complementary sources of biological evidence.

From this perspective, ESM-ECForest is perhaps best viewed as a prioritization tool rather than a definitive annotation system. The predicted EC assignments can be used to guide subsequent analyses and to generate hypotheses regarding enzyme function, particularly for poorly characterized proteins. Integration with transcriptomic, proteomic, metabolomic, phenotypic, and curated biochemical databases will remain necessary before such predictions can be incorporated into high-confidence metabolic reconstructions. Nevertheless, improvements in sequence-based enzyme annotation may help narrow the gap between the rapidly growing number of available protein sequences and the slower accumulation of experimentally validated metabolic knowledge.

### Limitations and future directions

One limitation of the current framework arises from the EC system itself. Although EC numbers provide a standardized description of enzyme activity, they do not always capture the full functional diversity of enzymes [3,4]. Proteins sharing the same EC assignment may differ substantially in substrate preference, cofactor dependence, reaction conditions, cellular localization, or physiological role. Conversely, a single EC designation may encompass multiple closely related biochemical reactions. As a result, accurate EC prediction does not necessarily translate into reaction-level resolution, which is often required for metabolic reconstruction and functional interpretation. Recent reaction-level modeling approaches address this limitation more directly. For example, ReactZyme uses a Swiss-Prot/Rhea benchmark for enzyme-reaction retrieval, formulating enzyme annotation as the prediction of specific biochemical reactions rather than EC numbers alone. These approaches suggest a potential direction for extending EC-based annotation frameworks toward finer-grained functional characterization [56,60,61].

This limitation highlights a broader challenge in enzyme annotation. Future developments are likely to require representations that extend beyond EC classification alone and incorporate additional layers of biochemical information [62]. Integrating substrate specificity, cofactor requirements, pathway context, cellular compartmentation, and reaction-level resources such as Rhea could help establish a more direct link between protein sequences and biochemical reactions [56]. Such efforts are consistent with recent attempts to move beyond traditional EC-based enzyme annotation and to represent enzyme function in a more flexible and biologically informative manner [17,34,38,62]. One example is the ECxit framework, which aims to redesign enzyme function annotation through a hierarchical deep-learning-based approach [63].

Several limitations of the present evaluation should also be considered. ESM-ECForest was trained and evaluated using selected prokaryotic and unicellular eukaryotic proteins from UniProtKB/Swiss-Prot, and its performance may not generalize equally well to other taxonomic groups (e.g., multicellular eukaryotes or human proteins) or poorly represented enzyme families. In addition, Stage B is restricted to the 3,021 terminal EC labels represented in the training dataset and cannot directly assign previously unseen terminal EC numbers. These constraints define the current taxonomic and functional scope of the model rather than its computational scalability for large microbial genomic and metagenomic datasets. However, higher-level EC annotations may still be recovered when predicted and reference EC numbers share the same higher-level classifications. In addition, the bootstrap CIs in this study were estimated by resampling individual proteins. Although the benchmark was constructed with sequence-redundancy control relative to the training data, cluster-based resampling could provide a more conservative assessment of uncertainty when related proteins are present in the benchmark dataset. Additionally, although the independent benchmark and stratified analyses provide a systematic assessment of predictive performance, they do not fully establish the model’s practical utility in biological annotation workflows. Future studies incorporating case studies of experimentally characterized proteins could provide additional insights into individual predictions, particularly in challenging or biologically relevant annotation scenarios.

A further limitation concerns the definition of the Stage A negative class. Proteins lacking EC and catalytic-activity annotations were used as operational nonenzyme examples, consistent with previous enzyme-prediction studies, but the absence of such annotations does not prove absence of catalytic activity. Our subsequent re-evaluation confirmed that while the majority of these proteins had annotations consistent with noncatalytic functions, a subset showed current or tentative evidence of enzyme activity and a further fraction remained uncertain. Stage A performance should therefore be interpreted in the context of this unavoidable label uncertainty rather than as classification against a set of experimentally validated nonenzymes. Furthermore, the positive enzyme class includes UniProt annotations with potentially different levels of experimental support, including computationally inferred functions. Since the experimental-evidence composition of this class was not systematically quantified, the reported performance should be interpreted in the context of annotation-derived labels rather than as a direct measure of experimentally validated catalytic activity.

Another limitation concerns potential exposure of benchmark sequences during PLM pretraining. Although benchmark proteins were excluded from all downstream supervised training, validation, and test data used to develop ESM-ECForest, ESM-2 itself was pretrained on a large external protein sequence corpus. We therefore cannot establish that benchmark proteins, or close homologs, were entirely unseen during PLM pretraining. This is distinct from supervised label leakage, but it limits the extent to which the benchmark can be interpreted as evaluation on sequence space completely unseen by the pretrained representation model. A temporally defined benchmark assembled from proteins deposited after the ESM-2 pretraining period could provide a useful complementary evaluation in future work, although such a design would still not exclude exposure to related homologs during pretraining.

There is also scope for methodological improvements. Although the current framework performs well across multiple EC levels, the confidence associated with individual predictions remains difficult to interpret. Improved probability calibration may be particularly valuable in applications where predictions are used to prioritize experimental validation or support manual curation. In addition, the persistent difficulty of classes such as EC 6 and EC 7 suggests that sequence information alone may not always be sufficient. Structural models, active-site annotations, transmembrane topology, domain organization, and protein-complex information could provide complementary evidence for distinguishing functionally similar enzymes [34].

Long proteins remain a limitation of the current framework. Sequences longer than 1,000 amino acids are represented by averaging embeddings from nonoverlapping sequence chunks, which may not fully capture domain architecture and long-range dependencies. Consistent with this, EC Level 4 performance was lower for proteins >1,000 amino acids than for shorter proteins. More effective strategies for representing long proteins could further improve performance on this subset.

The choice of PLM represents an additional design consideration. We used the compact esm2_t6_8M_UR50D model to limit the computational cost of representation generation and to retain compatibility with large-scale sequence annotation workflows. Larger ESM-2 variants and more recent PLMs, including ESM-C, may provide richer sequence representations and could potentially improve predictive performance. However, systematically comparing alternative PLMs would require model-specific optimization of representation extraction, dimensionality, sequence-length handling, and computational settings and was beyond the scope of the present study. Accordingly, the current results should not be interpreted as demonstrating that the selected ESM-2 model is optimal for enzyme function prediction. Comparative evaluation of newer and larger PLMs represents an important direction for future work.

More broadly, the results underscore the value of pretrained protein representations for large-scale enzyme annotation. These results suggest that competitive EC-annotation performance can be achieved using pretrained protein representations coupled with a lightweight classifier. They should not be interpreted as evidence that random forest is inherently superior to alternative classification methods. Furthermore, differences in the size, composition, and release dates of the training datasets used by competing methods may contribute to the observed performance differences, which therefore cannot be attributed solely to the choice of protein representations or classification algorithms. At the same time, the remaining prediction errors indicate that enzyme function cannot always be inferred from sequence alone. A natural next step will be to combine sequence-derived representations with structural, biochemical, and systems-level information, thereby moving from EC prediction toward functionally resolved and model-ready metabolic annotations.

## Data Availability

All data supporting the findings of this study are available within the article and its Supplementary Materials. The source code required to reproduce the analyses is available at GitHub (https://github.com/Xiao88866/ESM-ECForest). The trained models generated during this study are publicly available through Zenodo (https://doi.org/10.5281/zenodo.21699471).
