# Supplementary material for: A Two-Stage ESM-Based Machine Learning Pipeline for Robust Hierarchically Evaluated Enzyme Function Prediction
Source: Comput Struct Biotechnol J. 2026 Oct 29;35(1):0246. doi: 10.34133/csbj.0246 (PMC13716986; doi:10.34133/csbj.0246)
Supplement: Supplementary file 1 [file csbj.0246.f1.zip › ESM-ECForest_Supplementary_revision_clean.docx]

**Supporting information for: A Two-Stage ESM-Based Machine Learning Pipeline for Robust Hierarchical Enzyme Function Prediction**

# Supporting Figures

Figure S1: Relationship between EC-label training support and benchmark F1 score. Each point is one EC label. The x-axis is the log10-transformed number of training proteins annotated with that EC; the y-axis is the corresponding per-EC F1 on the benchmark dataset.

Figure S2: EC Level 4 performance across sequence-identity bins. Proteins were grouped according to their maximum sequence identity to the training dataset. Macro- and weighted-average F1 scores were calculated for each bin.

# Supporting Tables

**Table S1:** Taxonomic groups included in dataset construction.

| **Organism group** | **Taxon ID** |
| --- | --- |
| Bacteria | 2 |
| Archaea | 2157 |
| Saccharomyces | 4930 |
| Schizosaccharomyces | 4895 |
| Tetrahymena | 5890 |
| Trypanosoma | 5690 |
| Leishmania | 5658 |
| Plasmodium | 5820 |
| Giardia | 5740 |
| Entamoeba | 5758 |
| Chlamydomonas | 3052 |
| Euglena | 3038 |
| Dunaliella | 3044 |
| Candida | 5475 |
| Cryptococcus | 5206 |

**Table S2A:** Stage-A predictions for the post hoc audit categories of the nominal non-enzyme set.

| Verification category | n | Predicted enzyme | Predicted non-enzyme | % predicted enzyme |
| --- | --- | --- | --- | --- |
| No_enzyme | 49,396 | 78 | 49,318 | 0.16% |
| Uncertain | 18,120 | 150 | 17,970 | 0.83% |
| Likely_enzyme | 8,418 | 37 | 8,381 | 0.44% |
| Enzyme | 4,432 | 67 | 4,365 | 1.51% |
| All | 80,366 | 332 | 80,034 | 0.41% |

**Table S2B:** Stage-A enzyme-probability distributions across post hoc verification categories.

| Verification category | n | Mean p | Median p | p95 |
| --- | --- | --- | --- | --- |
| No_enzyme | 49,396 | 0.0247 | 0.0001 | 0.1516 |
| Uncertain | 18,120 | 0.0633 | 0.015 | 0.2687 |
| Likely_enzyme | 8,418 | 0.0324 | 0.005 | 0.1744 |
| Enzyme | 4,432 | 0.0774 | 0.0225 | 0.3093 |
| All | 80,366 | 0.0371 | 0.0026 | 0.1985 |

* p95 indicates the 95th percentile

**Table S3A:** Distribution of enzyme and non-enzyme sequences in the training, validation, and test sets.

| **Class** | **Train** | **Val** | **Test** | **Total** |
| --- | --- | --- | --- | --- |
| Enzyme | 90,704 | 4,615 | 9,329 | 104,648 |
| Non-enzyme | 64,841 | 6,095 | 9,430 | 80,366 |

**Table S3B:** Distribution of EC level 1 annotations in the training, validation, and test sets.

| **EC class** | **Train** | **Val** | **Test** | **Total** |
| --- | --- | --- | --- | --- |
| 1 | 29,572 | 955 | 503 | 31,030 |
| 2 | 34,975 | 10,196 | 1,439 | 46,610 |
| 3 | 16,407 | 2,963 | 486 | 19,856 |
| 4 | 14,087 | 939 | 919 | 15,945 |
| 5 | 2,891 | 112 | 80 | 3,083 |
| 6 | 6,488 | 70 | 97 | 6,655 |
| 7 | 503 | 16 | 24 | 543 |

**Table S4:** Training-support distribution of Stage-B terminal EC labels

| Training positives per EC label | Number of EC labels | Percentage of EC labels |
| --- | --- | --- |
| 1 | 1,275 | 47.22% |
| 2–5 | 965 | 35.74% |
| 6–10 | 189 | 7.00% |
| 11–20 | 90 | 3.33% |
| 21–50 | 74 | 2.74% |
| 51–100 | 39 | 1.44% |
| 101–500 | 30 | 1.11% |
| >500 | 39 | 1.44% |

**Table S5:** Random Forest hyperparameters used in ESM-ECForest.

| **Hyperparameter** | **Stage A enzyme classifier** | **Stage B EC classifier** |
| --- | --- | --- |
| n_estimators | 400 | 400 for each binary classifier |
| max_depth | 40 | 40 |
| bootstrap | True | True |
| max_samples | 0.8 | 0.8 |
| min_samples_split | 3 | 3 |
| min_samples_leaf | 1 | 1 |
| max_features | ’sqrt’ | ‘sqrt’ |
| random_state | 42 | 42 |
| n_jobs | -1 | -1 |

**Table S6:** Performance of Stage B under different probability thresholds at EC Level 4.

| Threshold | Macro F1 | Weighted F1 | Macro precision | Macro recall |
| --- | --- | --- | --- | --- |
| 0.3 | 0.758 | 0.881 | 0.702 | 0.837 |
| 0.5 | 0.779 | 0.895 | 0.761 | 0.812 |
| 0.7 | 0.79 | 0.902 | 0.808 | 0.785 |
| 0.9 | 0.751 | 0.874 | 0.866 | 0.676 |

**Table S7:** Species included in the benchmarking dataset.

| **Species** | **Taxon ID** |
| --- | --- |
| *Yarrowia lipolytica* | 4952 |
| *Komagataella pastoris* | 4922 |
| *Debaryomyces hansenii* | 4959 |
| *Dictyostelium discoideum* | 44689 |
| *Acanthamoeba castellanii* | 5755 |
| *Toxoplasma gondii* | 5811 |
| *Paramecium tetraurelia* | 5888 |
| *Naegleria gruberi* | 5762 |
| *Micromonas pusilla* | 38833 |
| *Corynebacterium glutamicum* | 1718 |
| *Deinococcus radiodurans* | 243230 |
| *Shewanella oneidensis* | 211586 |
| *Methanosarcina mazei* | 192952 |
| *Nitrosopumilus maritimus* | 436308 |
| *Thermococcus kodakarensis* | 69014 |

**Table S8:** Software implementations used for benchmarking.

| **Tool** | **Implementation** | **Commit** |
| --- | --- | --- |
| DeepEC | Bitbucket | adf79c9571d52218302c5323835f3508d88361aa |
| ProteInfer | GitHub | 0acc7b16eb93f21d88c15dcfe3c7828aef2881c8 |
| DeepECTransformer | GitHub | 0eff1da154a31ad2b20909bd4bb48fbf6d55c569 |

**Table S9:** Hardware and software specifications of the benchmarking environment.

| **Component** | **Specification** |
| --- | --- |
| CPU | AMD EPYC 7413 24-Core Processor |
| CPU threads used | 24 |
| Memory allocated | 200 GB |
| GPU | Not used |
| Python | 3.9.23 |
| PyTorch | 2.7.1+cu126 |

**Table S10:** Performance metrics of Stage A binary enzyme identification

| **Metric** | **Estimate** | **95% CI** |
| --- | --- | --- |
| Precision | 0.975 | [0.972, 0.977] |
| Recall | 0.977 | [0.975, 0.980] |
| F1 | 0.976 | [0.974, 0.978] |

Supplementary Table S11 is provided as a separate Excel file named Supplementary_table_S11_enzyme_vs_non-enzyme_re-annotation.xlsx.

**Table S12A:** Hierarchical EC prediction performance of ESM-ECForest across EC Levels 1–4.

| **Metric** | **EC level** | **Macro (95% CI)** | **Micro (95% CI)** | **Weighted (95% CI)** |
| --- | --- | --- | --- | --- |
| F1 | 1 | 0.911 [0.902, 0.919] | 0.943 [0.940, 0.946] | 0.944 [0.941, 0.947] |
| F1 | 2 | 0.847 [0.837, 0.855] | 0.921 [0.917, 0.924] | 0.929 [0.925, 0.934] |
| F1 | 3 | 0.826 [0.815, 0.835] | 0.912 [0.908, 0.916] | 0.920 [0.915, 0.924] |
| F1 | 4 | 0.790 [0.778, 0.799] | 0.888 [0.883, 0.892] | 0.902 [0.896, 0.906] |
| Precision | 1 | 0.896 [0.886, 0.904] | 0.952 [0.948, 0.956] | 0.953 [0.949, 0.957] |
| Precision | 2 | 0.864 [0.853, 0.874] | 0.924 [0.918, 0.929] | 0.943 [0.938, 0.947] |
| Precision | 3 | 0.848 [0.837, 0.859] | 0.911 [0.904, 0.917] | 0.932 [0.926, 0.937] |
| Precision | 4 | 0.808 [0.796, 0.819] | 0.882 [0.875, 0.889] | 0.910 [0.903, 0.915] |
| Recall | 1 | 0.928 [0.919, 0.935] | 0.935 [0.932, 0.938] | 0.935 [0.932, 0.938] |
| Recall | 2 | 0.842 [0.832, 0.851] | 0.918 [0.914, 0.922] | 0.918 [0.914, 0.922] |
| Recall | 3 | 0.821 [0.811, 0.832] | 0.913 [0.909, 0.917] | 0.913 [0.909, 0.917] |
| Recall | 4 | 0.785 [0.773, 0.796] | 0.895 [0.890, 0.899] | 0.895 [0.891, 0.899] |

**Table S12B:** Class-specific EC prediction performance of ESM-ECForest at EC Level 1.

| **EC class** | **Precision** | **Recall** | **F1 score** | **Support** |
| --- | --- | --- | --- | --- |
| 1 | 0.983 | 0.948 | 0.965 | 4663 |
| 2 | 0.944 | 0.920 | 0.932 | 3212 |
| 3 | 0.973 | 0.939 | 0.956 | 4914 |
| 4 | 0.874 | 0.908 | 0.891 | 878 |
| 5 | 0.854 | 0.945 | 0.897 | 629 |
| 6 | 0.828 | 0.896 | 0.861 | 366 |
| 7 | 0.815 | 0.940 | 0.873 | 185 |

**Table S13:** Protein-level multi-label EC prediction performance at EC Level 4.

| Protein group | n proteins | Sample precision | Sample recall | Sample F1 | Jaccard similarity | Exact-set accuracy |
| --- | --- | --- | --- | --- | --- | --- |
| All enzymes | 14,847 | 0.877 | 0.907 | 0.886 | 0.876 | 0.844 |
| Single-EC proteins | 7,319 | 0.96 | 0.972 | 0.963 | 0.958 | 0.934 |
| Multifunctional proteins | 7,528 | 0.792 | 0.841 | 0.806 | 0.792 | 0.752 |

**Table S14:** Sequence-identity-stratified EC Level-4 performance.

| Max identity to training | n | Macro F1 | Weighted F1 |
| --- | --- | --- | --- |
| No detectable hit | 943 | 0.692 | 0.769 |
| <10% | 1,074 | 0.735 | 0.859 |
| 10–20% | 1,351 | 0.764 | 0.873 |
| 20-30% | 3,748 | 0.789 | 0.912 |
| 30–40% | 7,731 | 0.814 | 0.924 |

**Table S15A:** EC Level-4 performance stratified by protein length.

| Protein length group | n proteins | Macro F1 | Weighted F1 |
| --- | --- | --- | --- |
| ≤1,000 aa | 13,961 | 0.792 | 0.905 |
| >1,000 aa | 886 | 0.724 | 0.863 |

**Table S15B:** Distribution of proteins longer than 1,000 amino acids across datasets.

| Dataset / subset | ≤1,000 aa | >1,000 aa | % >1,000 aa |
| --- | --- | --- | --- |
| Training proteins | 183,476 | 1,538 | 0.83% |
| Benchmark proteins | 24,219 | 1,559 | 6.05% |
| Benchmark enzymes | 13,961 | 886 | 5.97% |

**Table S15C:** EC Level-1 composition of benchmark enzymes longer than 1,000 amino acids.

| EC class | n long proteins | % of long proteins |
| --- | --- | --- |
| EC1 | 206 | 23.25% |
| EC2 | 456 | 51.47% |
| EC3 | 310 | 34.99% |
| EC4 | 181 | 20.43% |
| EC5 | 125 | 14.11% |
| EC6 | 128 | 14.45% |
| EC7 | 32 | 3.61% |

*Percentages were calculated from the 886 benchmark enzymes longer than 1,000 amino acids. Because some enzymes are associated with multiple EC Level-1 classes, the percentages do not add up to 100%.

**Table S15D:** Annotation multiplicity of benchmark enzymes stratified by protein length.

| Length group | Single-EC | Multifunctional |
| --- | --- | --- |
| ≤1,000 aa | 6,852 | 7,109 |
| >1,000 aa | 467 | 419 |

**Table S16A:** Two-stage gate ablation (Level 4)

| Setting | Description | Macro F1 | Weighted F1 |
| --- | --- | --- | --- |
| Current ESM-ECForest | Stage A predicted enzymes passed to Stage B | 0.79 | 0.902 |
| Oracle gate + Stage B | True enzyme status used as gate | 0.858 | 0.921 |
| No gate + Stage B | Stage B applied without Stage A filtering | 0.781 | 0.863 |

**Table S16B:** Representation and classifier ablation(Level 4)

| Ablation | Embedding | Classifier | Macro F1 | Weighted F1 |
| --- | --- | --- | --- | --- |
| Main model | BOS | Random Forest | 0.79 | 0.902 |
| Representation ablation | Mean pooling | Random Forest | 0.784 | 0.916 |
| Classifier ablation | BOS | Logistic Regression | 0.374 | 0.815 |
| Classifier ablation | BOS | GBM | 0.736 | 0.901 |

*All ablations were evaluated on the same independent benchmark dataset at EC Level 4. BOS, beginning-of-sequence token embedding from the final ESM-2 layer; mean pooling, average of residue-level ESM-2 embeddings; GBM, Gradient Boosting Machine.

**Table S17A:** Performance of ESM-ECForest and comparison methods across the four levels of the EC hierarchy. Weighted F1, Micro F1, and Macro F1 scores are reported together with their 95% bootstrap confidence intervals (CI).

| **Metric** | **EC level** | **Weighted F1 (95% CI)** | **Micro F1 (95% CI)** | **Macro F1 (95% CI)** |
| --- | --- | --- | --- | --- |
| ESM-ECForest | 1 | 0.944 [0.941, 0.947] | 0.943 [0.940, 0.946] | 0.911 [0.902, 0.919] |
|  | 2 | 0.929 [0.925, 0.934] | 0.921 [0.917, 0.924] | 0.847 [0.837, 0.855] |
|  | 3 | 0.920 [0.915, 0.924] | 0.912 [0.908, 0.916] | 0.826 [0.815, 0.835] |
|  | 4 | 0.902 [0.896, 0.906] | 0.888 [0.883, 0.892] | 0.790 [0.778, 0.799] |
| DeepECTransformer | 1 | 0.925 [0.920, 0.930] | 0.925 [0.920, 0.930] | 0.889 [0.878, 0.900] |
|  | 2 | 0.907 [0.901, 0.913] | 0.906 [0.900, 0.912] | 0.854 [0.841, 0.867] |
|  | 3 | 0.889 [0.883, 0.895] | 0.887 [0.881, 0.893] | 0.815 [0.800, 0.830] |
|  | 4 | 0.857 [0.850, 0.864] | 0.854 [0.847, 0.861] | 0.758 [0.740, 0.776] |
| ProteInfer | 1 | 0.918 [0.913, 0.923] | 0.919 [0.914, 0.923] | 0.863 [0.851, 0.875] |
|  | 2 | 0.890 [0.884, 0.896] | 0.890 [0.884, 0.896] | 0.821 [0.807, 0.835] |
|  | 3 | 0.873 [0.867, 0.879] | 0.871 [0.865, 0.877] | 0.783 [0.767, 0.799] |
|  | 4 | 0.821 [0.813, 0.829] | 0.817 [0.809, 0.825] | 0.699 [0.678, 0.720] |
| DeepEC | 1 | 0.660 [0.652, 0.668] | 0.680 [0.672, 0.688] | 0.559 [0.541, 0.577] |
|  | 2 | 0.631 [0.622, 0.640] | 0.644 [0.635, 0.653] | 0.511 [0.492, 0.530] |
|  | 3 | 0.602 [0.592, 0.612] | 0.611 [0.601, 0.621] | 0.462 [0.442, 0.482] |
|  | 4 | 0.548 [0.537, 0.559] | 0.553 [0.542, 0.564] | 0.389 [0.367, 0.411] |
| MMseqs2 | 1 | 0.705 [0.701, 0.709] | 0.706 [0.702, 0.711] | 0.622 [0.615, 0.629] |
|  | 2 | 0.635 [0.631, 0.638] | 0.649 [0.645, 0.652] | 0.442 [0.434, 0.451] |
|  | 3 | 0.589 [0.585, 0.593] | 0.592 [0.588, 0.596] | 0.419 [0.411, 0.429] |
|  | 4 | 0.503 [0.499, 0.507] | 0.514 [0.510, 0.519] | 0.301 [0.289, 0.310] |
| CLEAN | 1 | 0.951 [0.948, 0.955] | 0.950 [0.948, 0.956] | 0.923 [0.916, 0.929] |
|  | 2 | 0.934 [0.930, 0.938] | 0.929 [0.926, 0.933] | 0.899 [0.892, 0.908] |
|  | 3 | 0.916 [0.911, 0.921] | 0.918 [0.914, 0.922] | 0.813 [0.804, 0.823] |
|  | 4 | 0.907 [0.901, 0.914] | 0.911 [0.905, 0.916] | 0.786 [0.776, 0.797] |

* MMseqs2 denotes MMseqs2 nearest-neighbor transfer.

**Table S17B:** Class-specific EC prediction performance of DeepECTransformer at EC Level 1.

| **EC class** | **Precision** | **Recall** | **F1 score** | **Support** |
| --- | --- | --- | --- | --- |
| 1 | 0.948 | 0.936 | 0.942 | 4663 |
| 2 | 0.922 | 0.908 | 0.915 | 3212 |
| 3 | 0.941 | 0.929 | 0.935 | 4914 |
| 4 | 0.918 | 0.862 | 0.889 | 878 |
| 5 | 0.958 | 0.881 | 0.918 | 629 |
| 6 | 0.864 | 0.827 | 0.845 | 366 |
| 7 | 0.799 | 0.754 | 0.776 | 185 |

**Table S17C:** Class-specific EC prediction performance of ProteInfer at EC Level 1.

| **EC class** | **Precision** | **Recall** | **F1 score** | **Support** |
| --- | --- | --- | --- | --- |
| 1 | 0.942 | 0.935 | 0.938 | 4663 |
| 2 | 0.915 | 0.894 | 0.904 | 3212 |
| 3 | 0.935 | 0.943 | 0.939 | 4914 |
| 4 | 0.890 | 0.841 | 0.865 | 878 |
| 5 | 0.904 | 0.849 | 0.876 | 629 |
| 6 | 0.829 | 0.799 | 0.814 | 366 |
| 7 | 0.733 | 0.673 | 0.702 | 185 |

**Table S17D:** Class-specific EC prediction performance of DeepEC at EC Level 1.

| **EC class** | **Precision** | **Recall** | **F1 score** | **Support** |
| --- | --- | --- | --- | --- |
| 1 | 0.818 | 0.783 | 0.800 | 4663 |
| 2 | 0.762 | 0.729 | 0.745 | 3212 |
| 3 | 0.832 | 0.364 | 0.506 | 4914 |
| 4 | 0.683 | 0.618 | 0.649 | 878 |
| 5 | 0.718 | 0.632 | 0.672 | 629 |
| 6 | 0.586 | 0.502 | 0.541 | 366 |
| 7 | - | - | - | 185* |

*DeepEC does not provide EC 7 predictions. EC 7 was retained in the overall hierarchical evaluation as an unsupported class but is shown as N/A in class-specific evaluation.

**Table S17E:** Class-specific EC prediction performance of CLEAN at EC Level 1.

| **EC class** | **Precision** | **Recall** | **F1 score** | **Support** |
| --- | --- | --- | --- | --- |
| 1 | 0.972 | 0.964 | 0.968 | 4,663 |
| 2 | 0.958 | 0.944 | 0.951 | 3,212 |
| 3 | 0.951 | 0.937 | 0.944 | 4,914 |
| 4 | 0.94 | 0.95 | 0.945 | 878 |
| 5 | 0.968 | 0.95 | 0.959 | 629 |
| 6 | 0.901 | 0.878 | 0.889 | 366 |
| 7 | 0.821 | 0.796 | 0.808 | 185 |

**Table S17F:** Class-specific EC prediction performance of MMseqs2 nearest-neighbor transfer at EC Level 1.

| **EC class** | **Precision** | **Recall** | **F1 score** | **Support** |
| --- | --- | --- | --- | --- |
| 1 | 0.793 | 0.748 | 0.77 | 4,663 |
| 2 | 0.704 | 0.637 | 0.669 | 3,212 |
| 3 | 0.728 | 0.663 | 0.694 | 4,914 |
| 4 | 0.773 | 0.717 | 0.744 | 878 |
| 5 | 0.747 | 0.693 | 0.719 | 629 |
| 6 | 0.483 | 0.387 | 0.43 | 366 |
| 7 | 0.376 | 0.293 | 0.329 | 185 |

**Table S18:** Common-label comparison at EC Level 4 restricted to shared EC classes EC1–EC6.

| Method | F1 micro | F1 macro | F1 weighted |
| --- | --- | --- | --- |
| ESM-ECForest | 0.889 | 0.801 | 0.903 |
| CLEAN | 0.913 | 0.791 | 0.908 |
| MMseqs2-1NN | 0.521 | 0.326 | 0.510 |
| ProteInfer | 0.818 | 0.701 | 0.823 |
| DeepEC | 0.564 | 0.469 | 0.565 |
| DeepECtransformer | 0.857 | 0.763 | 0.859 |

**Table S19:** Benchmark EC labels absent from Stage-B training

| **Quantity** | **Value** |
| --- | --- |
| Distinct benchmark Level-4 EC labels | 2,506 |
| Labels absent from Stage-B training set | 481 |
| Percentage absent from training | 19.18% |
